# Insights into malaria transmission among *Anopheles funestus* mosquitoes, Kenya

**DOI:** 10.1186/s13071-018-3171-3

**Published:** 2018-11-06

**Authors:** Edwin O. Ogola, Ulrike Fillinger, Isabella M. Ondiba, Jandouwe Villinger, Daniel K. Masiga, Baldwyn Torto, David P. Tchouassi

**Affiliations:** 1 International Centre of Insect Physiology and Ecology (icipe), Nairobi, Kenya; 2 School of Biological Sciences, University of Nairobi, Nairobi, Kenya

**Keywords:** *Anopheles funestus* group, *Anopheles longipalpis* C, Malaria parasite transmission, Molecular approaches, Entomological surveillance, Kenya

## Abstract

**Background:**

Most malaria vectors belong to species complexes. Sibling species often exhibit divergent behaviors dictating the measures that can be deployed effectively in their control. Despite the importance of the *Anopheles funestus* complex in malaria transmission in sub-Saharan Africa, sibling species have rarely been identified in the past and their vectoring potential remains understudied.

**Methods:**

We analyzed 1149 wild-caught *An. funestus* (*senso lato*) specimens from 21 sites in Kenya, covering the major malaria endemic areas including western, central and coastal areas. Indoor and outdoor collection tools were used targeting host-seeking and resting mosquitoes. The identity of sibling species, infection with malaria *Plasmodium* parasites, and the host blood meal sources of engorged specimens were analyzed using PCR-based and sequencing methods.

**Results:**

The most abundant sibling species collected in all study sites were *Anopheles funestus* (59.8%) and *Anopheles rivulorum* (32.4%) among the 1062 successfully amplified specimens of the *An. funestus* complex. Proportionally, *An. funestus* dominated in indoor collections whilst *An. rivulorum* dominated in outdoor collections. Other species identified were *Anopheles leesoni* (4.6%), *Anopheles parensis* (2.4%), *Anopheles vaneedeni* (0.1%) and for the first time in Kenya, *Anopheles longipalpis* C (0.7%). *Anopheles funestu*s had an overall *Plasmodium* infection rate of 9.7% (62/636), predominantly *Plasmodium falciparum* (59), with two infected with *Plasmodium ovale* and one with *Plasmodium malariae*. There was no difference in the infection rate between indoor and outdoor collections. Out of 344 *An. rivulorum*, only one carried *P. falciparum.* We also detected *P. falciparum* infection in two non-blood-fed *An. longipalpis* C (2/7) which is the first record for this species in Kenya. The mean human blood indices for *An. funestus* and *An. rivulorum* were 68% (93/136) and 64% (45/70), respectively, with feeding tendencies on a broad host range including humans and domestic animals such as cow, goat, sheep, chicken and pig.

**Conclusions:**

Our findings underscore the importance of active surveillance through application of molecular approaches to unravel novel parasite-vector associations possibly contributed by cryptic species with important implications for effective malaria control and elimination.

**Electronic supplementary material:**

The online version of this article (10.1186/s13071-018-3171-3) contains supplementary material, which is available to authorized users.

## Background

Malaria remains the most important vector-borne disease in tropical countries of the world, especially in sub-Saharan Africa (SSA). The scale-up of long lasting insecticidal net (LLIN) distribution has contributed to a decline in malaria transmission, number of cases and mortality within the last decade [1], prompting efforts toward a target of 2030 for elimination. Sustained malaria reduction has proved challenging in many disease-endemic countries of Africa. Whilst in many settings LLIN coverage is high, malaria transmission persists [2] and there is need for a better understanding of the vector species composition. Surveillance of vector populations becomes increasingly important to inform fine-tuning of control strategies in the event of changing local conditions [3] or to discern cryptic species contributing to stealth transmission.

The major African malaria vectors belong to two species complexes: *Anopheles gambiae* and *Anopheles funestus* [4, 5]. Few studies in Kenya have examined the contribution of *Anopheles funestus* sibling species to malaria transmission, despite its well-established role in malaria transmission [6]. It is well known that *An. funestus* (*sensu stricto*) (hereafter referred to as *An. funestus*) is the primary vector species in the *An. funestus* group [5]. Recent reports of novel *Plasmodium falciparum* sporozoite associations in *An. vaneedeni* [7], as well as yet-to-be-identified species [8, 9], suggest the possibility of adaptive changes in vector systems among the group. This underscores the importance of active surveillance of vector populations for potential emerging threats as malaria is being controlled.

Bionomic traits and susceptibility to *Plasmodium* infection vary among the 13 sibling species known to occur throughout the Afrotropical region in the *An. funestus* group: *An. funestus*, *An. funestus-*like, *An. aruni*, *An. confusus*, *An. parensis*, *An. vaneedeni*, *An. longipalpis* types A and C, *An. leesoni*, *An. rivulorum*, *An. rivulorum-*like, *An. brucei* and *An. fuscivenosus* [5]. Five species have been documented to occur in Kenya, namely *An. funestus*, *An. rivulorum*, *An. leesoni*, *An. parensis* and *An. vaneedeni* [6, 10]. Of these, *P. falciparum* infection has been found in *An. funestus* as the main vector [11, 12] and to a minor extent in *An. rivulorum* [13, 14] and *An. leesoni* [9]. The often small sample sizes of *An. funestus* (*s.l.*) mosquitoes analyzed by molecular means in most of these studies preclude a detailed understanding of the occurrence and ecological distribution of member species in this complex and corollary, their vectoring potential of malaria parasites.

This study focused on the *An. funestus* group in relation to possible ongoing variations in vector-parasite associations in Kenya. Specifically, we examined the identity and occurrence in the *An. funestus* group sibling species, *Plasmodium* infection rates and blood-feeding patterns from specimens sampled from varied malaria-endemic locations in coastal, western and Rift Valley areas of Kenya.

## Methods

### *Anopheles* samples

**Fig. 1:**
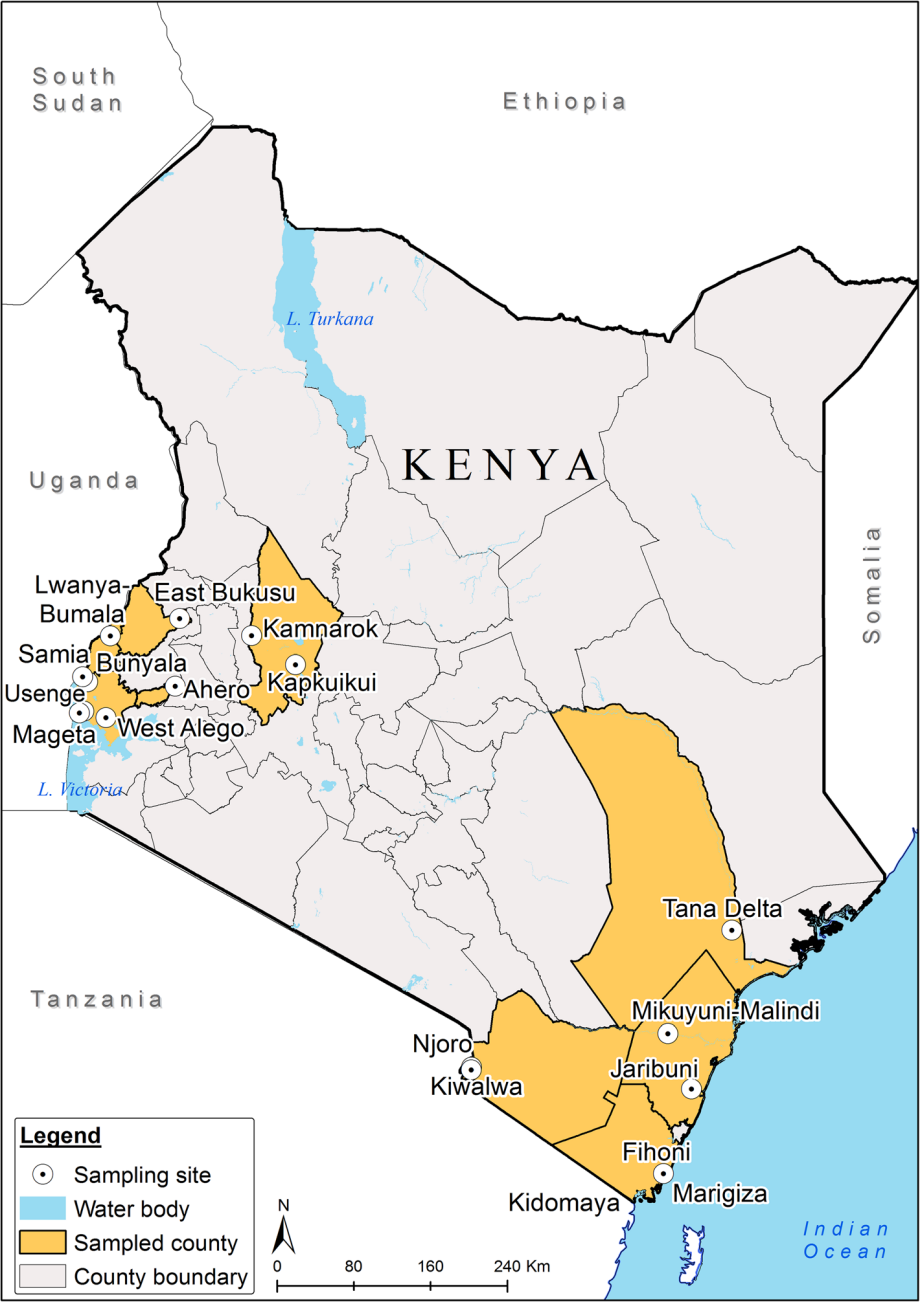
Map of Kenya showing the sampling locations

We analysed female specimens of the *An. funestus* complex collected from randomly selected consenting households in diverse sites in the different epidemiological risk zones for malaria in Kenya [15]. These included Kwale County (Kidomaya, Marigiza, Fihoni), Kilifi County (Jaribuni, Malindi), Taita-Taveta County (Kiwalwa, Njoro), Tana River County (Tana Delta) in the coastal region and Busia County (Bunyala, East Bukusu, Samia, Lwanya-Bumala), Kisumu County (Ahero) and Siaya County (Usenge, West Alego, Mageta) in the Lake Victoria basin in western Kenya and more seasonal malaria transmission areas including the arid and semi-arid areas of northern and central Kenya such as Baringo County (Kapkuikui, Kamnarok) (Fig. 1). Samples from the study sites were mainly collected in 2017, except for those from Ahero, Usenge and Kamnarok (2015), and Mageta (2014).

Mosquito collections were undertaken both indoors and outdoors with CDC light traps targeting host-seeking mosquitoes for most sites (Bunyala, East Bukusu, Samia, Lwanya-Bumala, West Alego, Kidomaya, Marigiza, Fihoni, Jaribuni). This was achieved by deploying 10 traps per site (5 indoors and 5 outdoors) in separate randomly selected households. At the sites in Ahero, Tana Delta, Kapkuikui, collections were limited to outdoors only using CDC light traps (10) while in Kamnarok, Usenge, Mageta, Njoro and Kiwalwa, collections were limited to indoors using pyrethrum spray catches (PSC) or backpack aspiration (ASP) targeting resting mosquitoes. CDC light traps were set at 18:00 h and retrieved at 06:00 h the following day with PSC and ASP collections conducted between 06:00 and 08:00 h. Indoor and outdoor catches were not undertaken systematically to compare trapping efficiency indoors and outdoors at a particular site, but different tools and locations were used in an attempt to identify the mosquito species richness.

### DNA extraction and identification of *Anopheles funestus* sibling species

We extracted genomic DNA from individual whole specimens using the Qiagen DNeasy Blood and Tissue Kit (Qiagen, Hilden, Germany) as per the manufacturer’s instructions. The DNA was used for identification of the sibling species as well as probing for *Plasmodium* infection and host blood meal sources.

Molecular identification of member species of the *An. funestus* complex involved polymerase chain reaction (PCR) amplification of the polymorphic ITS2 region of ribosomal DNA using established primers [16, 17]. The PCR in a 15 μl reaction involved 0.5 μM each of the primers, 3 μl of 5× Hot Firepol Blend Master Mix Ready to Load (Solis BioDyne, Tartu, Estonia) and 2 μl of DNA template. PCR cycling conditions were as follows: initial denaturation at 95 °C for 15 min, followed by 30 cycles of denaturation at 95 °C for 30 s, annealing at 46 °C for 30 s and extension at 72°C for 40 s, and a final extension at 72 °C for 10 min. Thermal reactions were conducted on SimpliAmp Thermal Cycler (Applied Biosystems, Loughborough, UK). Size fragments characteristic of each species were distinguished after separation in agarose gel electrophoresis (1.5%) stained with ethidium bromide against a 100 bp DNA ladder (O’ Gene Ruler, Fermentas, Fisher Scientific, Loughborough, UK).

### PCR detection of *Plasmodium* malaria parasites

We screened for *Plasmodium* infection in individual specimens by analyzing high resolution melting (HRM) profiles generated from real time-PCR (RT-PCR-HRM) products of non-coding mitochondrial sequence (ncMS) as described before [18] and/or amplification of the cytochrome *c* oxidase 1 (*cox*1) gene [19]. *Plasmodium falciparum* DNA obtained from the National Institute for Biological Standards and Control (NIBSC; London, UK) was used as a standard reference positive control. RT-PCR-HRM was performed in RotorGene Q thermocycler (Qiagen) with RotorGene Q software v.2.1 (Qiagen) with *Plasmodium* infection determined by comparing melt curves to those obtained using a positive control. Conventional PCR for *Plasmodium* parasite detection targeting the *cox*1 gene was carried out in a 15 μl reaction containing 0.5 μM of each primer, 9 μl of PCR water, 3 μl of 5× Hot Firepol® Blend Master Mix (Solis BioDyne) and 2 μl of DNA template. Amplification was carried out with the following cycling parameters: 95 °C for 15 min, 40 cycles of 95 °C for 30 s, 59 °C for 30 s and 72 °C for 40 s, followed by 72 °C for 10 min. For further confirmation, the PCR product of all positive samples by RT-PCR-HRM and a subset of *cox*1 gene amplicons were purified using ExoSAP-IT (USB Corporation, Cleveland, OH, USA) and outsourced for sequencing (Macrogen, Seoul, Republic of Korea or University of Notre Dame, Notre Dame, Indiana, USA).

Sequences were viewed and edited in Chromas, embedded in MEGA v.6 software [20] prior to querying GenBank using BLAST. Multiple sequence alignments of the resulting contiguous sequences were performed using ClustalW with default parameters. Maximum likelihood phylogeny was computed using the best fit model of sequence evolution with nodal support for the different groupings evaluated through 1000 bootstrap resampling.

We further confirmed the species identity of *Plasmodium*-positive mosquito specimens by amplifying and sequencing of ribosomal DNA internal transcribed spacer region 2 (rDNA ITS2) gene [21] and/or the mitochondrial cytochrome *c* oxidase subunit 1 (*cox*1) gene [22]. PCR volumes for rDNA ITS2 and *cox*1 were: 15 μl containing 0.5 μM final concentrations for each primer, 9 μl of PCR water, 3 μl of 5× Hot Firepol Blend Master Mix (Solis BioDyne) and 2 μl of DNA template. Amplification was undertaken with the following cycling parameters: initial denaturation at 95 °C for 15 min, followed by 40 cycles of denaturation at 95 °C for 30 s, 30 s annealing at 60 and 50 °C for rDNA ITS2 and *cox*1, respectively, and extension at 72 °C for 40 s, and a final extension at 72 °C for 10 min. Thermal reactions were conducted on SimpliAmp Thermal Cycler (Applied Biosystems). The samples were purified as previously described and outsourced for sequencing (DNA Sequencing Facility, University of Illinois at Urbana-Champaign, Champaign, IL, USA). Sequences were similarly cleaned and compared to GenBank database and consensus sequences aligned using ClustalW or MUSCLE in MEGA v.6 for *cox*1 and ITS, respectively. Maximum likelihood (ML) trees were constructed (1000 bootstraps) with inclusion of reference sequences as outgroups utilizing the GTR+G and Kimura-2 parameter model of sequence evolution for *cox*1 and ITS gene fragments, respectively, in MEGA v.6.

### Blood-meal analyses

To detect blood-meal host sources, cytochrome b (*cyt b*), *16S* ribosomal rRNA and cytochrome *c* oxidase subunit 1 (*cox*1) fragments were amplified from genomic DNA extracted from engorged *Anopheles* mosquitoes using RT-PCR-HRM (Rotor Gene Q thermocycler; Qiagen) and compared to profiles of known positive controls as previously described [23]. Extracted DNA from sugar-fed insectary-reared *Anopheles* mosquitoes served as negative controls. Known vertebrate DNA used in a previous study, namely cow, Swiss mouse, pig, goat, chicken and human, were included as standard reference positive controls. PCR volumes of 10 μl contained 0.5 μM concentrations of each primer, 6 μl of water, 2 μl of 5× Hot Firepol Evagreen HRM Mix (Solis BioDyne) and 1 μl of DNA template. Amplification for *cyt b* and *16S* rRNA primers was carried out as previously described [23]. High resolution melting profiles generated were analyzed using HRM analysis tools present in the RGQ software. Vertebrate hosts were determined through comparison of the *Anopheles* mosquitoes’ blood meal HRM melt profiles to those of the standard reference control species.

### Statistical analysis

We used Pearson’s Chi-square tests in R v.3.3.1 [24] to evaluate the difference in proportions at an α = 0.05 level of significance. We established the *Plasmodium* infection rate (IR) as the percentage of the number positive out of the total number examined. Overall proportions of mosquitoes and IR were compared indoors and outdoors using Pearson’s Chi-square test.

## Results

**Fig. 2:**
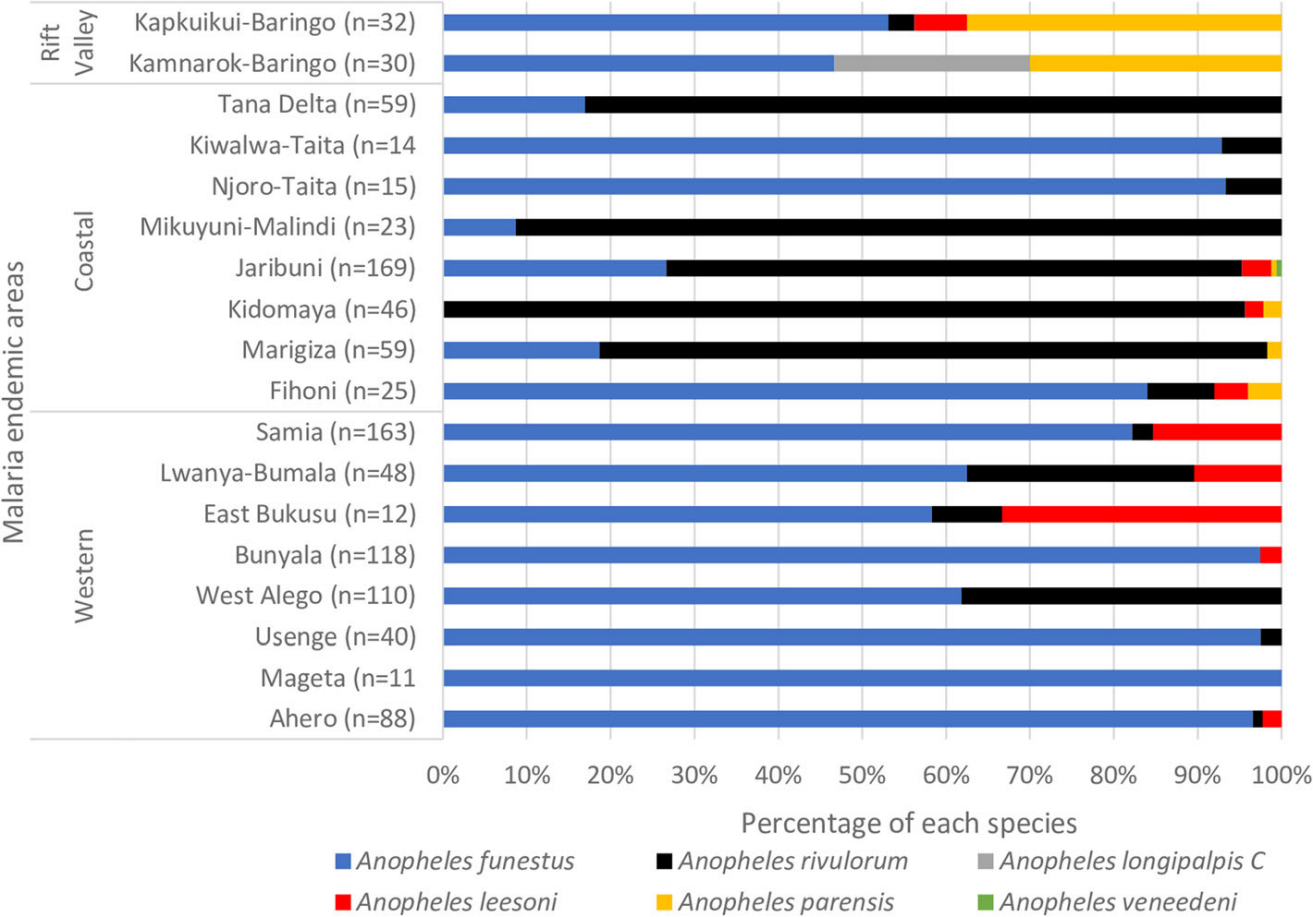
Relative frequency of species in the *Anopheles funestus* group, in the different malaria-endemic areas, Kenya. The numbers in parentheses indicate the total sample size of collected mosquitoes per site

We analyzed 1149 specimens of which 87 (7.6%) did not amplify in PCR. Of the 1062 that successfully amplified, 627 specimens originated from indoor sampling and 435 from outdoor sampling. *Anopheles funestus* was predominant (59.9%) followed by *An. rivulorum* (32.4%), *An. leesoni* (4.6%), *An. parensis* (2.4%), *An. longipalpis* C (0.7%) and *An. vaneedeni* (0.1%). Species composition of the *An. funestus* complex, however, varied between sampling areas, with higher proportions of *An. rivulorum* collected in coastal sites (Fig. 1). Proportionally, *An. funestus* dominated indoor collections (476/627; 75.9%), while specimens of *An. rivulorum* dominated in the outdoor collections, mainly from the coastal area (253/435; 58.2%) (Fig. 2, Table 1).

**Table 1:** Species composition of *Anopheles funestus* group and the species found positive for *Plasmodium* infections, Kenya

County	Site	*An. funestus*	*An. rivulorum*	*An. longipalpis* C	*An. leesoni*	*An. parensis*	*An. vaneedeni*
		No. positive (No. examined)	No. positive (No. examined)	No. positive (No. examined)	No. positive (No. examined)	No. positive (No. examined)	No. positive (No. examined)
Kisumu	Ahero	4 (85)	0 (1)	0	0 (2)	0	0
Siaya	Mageta	2 (11)	0	0	0	0	0
Siaya	Usenge	6 (39)	0 (1)	0	0	0	0
Siaya	West Alego	2 (68)	0 (42)	0	0	0	0
Busia	Bunyala	4 (115)	0	0	0 (3)	0	0
Busia	East Bukusu	2 (7)	0 (1)	0	0 (4)	0	0
Busia	Lwanya-Bumala	15 (30)	0 (13)	0	0 (5)	0	0
Busia	Samia	14 (134)	0 (4)	0	0 (25)	0	0
Kwale	Fihoni	2 (21)	0 (2)	0	0 (1)	0 (1)	0
Kwale	Marigiza	0 (11)	0 (47)	0	0	0 (1)	0
Kilifi	Kidomaya	0 (0)	0 (44)	0	0 (1)	0 (1)	0
Kilifi	Jaribuni	8 (45)	1 (116)	0	0 (6)	0 (1)	0 (1)
Kilifi	Mikuyuni-Malindi	0 (2)	0 (21)	0	0	0	0
Taita-Taveta	Njoro-Taita	0 (14)	0 (1)	0	0	0	0
Taita-Taveta	Kiwalwa-Taita	0 (13)	0 (1)	0	0	0	0
Baringo	Kamnarok-Baringo	2 (14)	0 (0)	2 (7)	0	9	0
Baringo	Kapkuikui-Baringo	0 (17)	0 (1)	0 (0)	0 (2)	0 (12)	0
Tana-River	Tana Delta	1 (10)	0 (49)	0	0	0	0
Total		62 (636)	1 (344)	2 (7)	0 (49)	0 (25)	0 (1)

Overall, we observed a significantly higher proportion of *An. funestus* indoors than outdoors (476/636 *vs* 160/636; *χ*^2^ = 156.0, *df* = 1, *P* < 0.001), confirming its highly endophilic nature. Likewise, captures of *An. leesoni* were significantly more frequent indoors than outdoors (40/49 *vs* 9/49; *χ*^2^ = 18.4, *df* = 1, *P* < 0.001). In contrast, a much higher proportion of *An. rivulorum* was trapped outdoors than indoors (253/344 *vs* 91/344; *χ*^2^ = 75.4, *df* = 1, *P* < 0.001) (Table 1) suggesting an exophilic tendency for this species. Comparable numbers of *An. parensis* were encountered indoors (12/25) and outdoors (13/25) (Fig 2, Table 1).

Sequencing of ncMS and/or *cox*1 gene amplicons indicated *Plasmodium* infections in 65 specimens, of which 62 shared 100% identity to *Plasmodium falciparum* [GenBank: CP017005 (ncMS); NC037526 (*cox*1)], two shared 100% identity to *P. ovale* [GenBank: AB354571 (ncMS); KP050417 (*cox*1)] and one shared 100% identity with *Plasmodium malariae* [MF693428, GQ355486 (*cox*1)]. These *Plasmodium* species identifications are supported by the sequence alignment (Additional file 1: Figure S1).

**Fig. 3:**
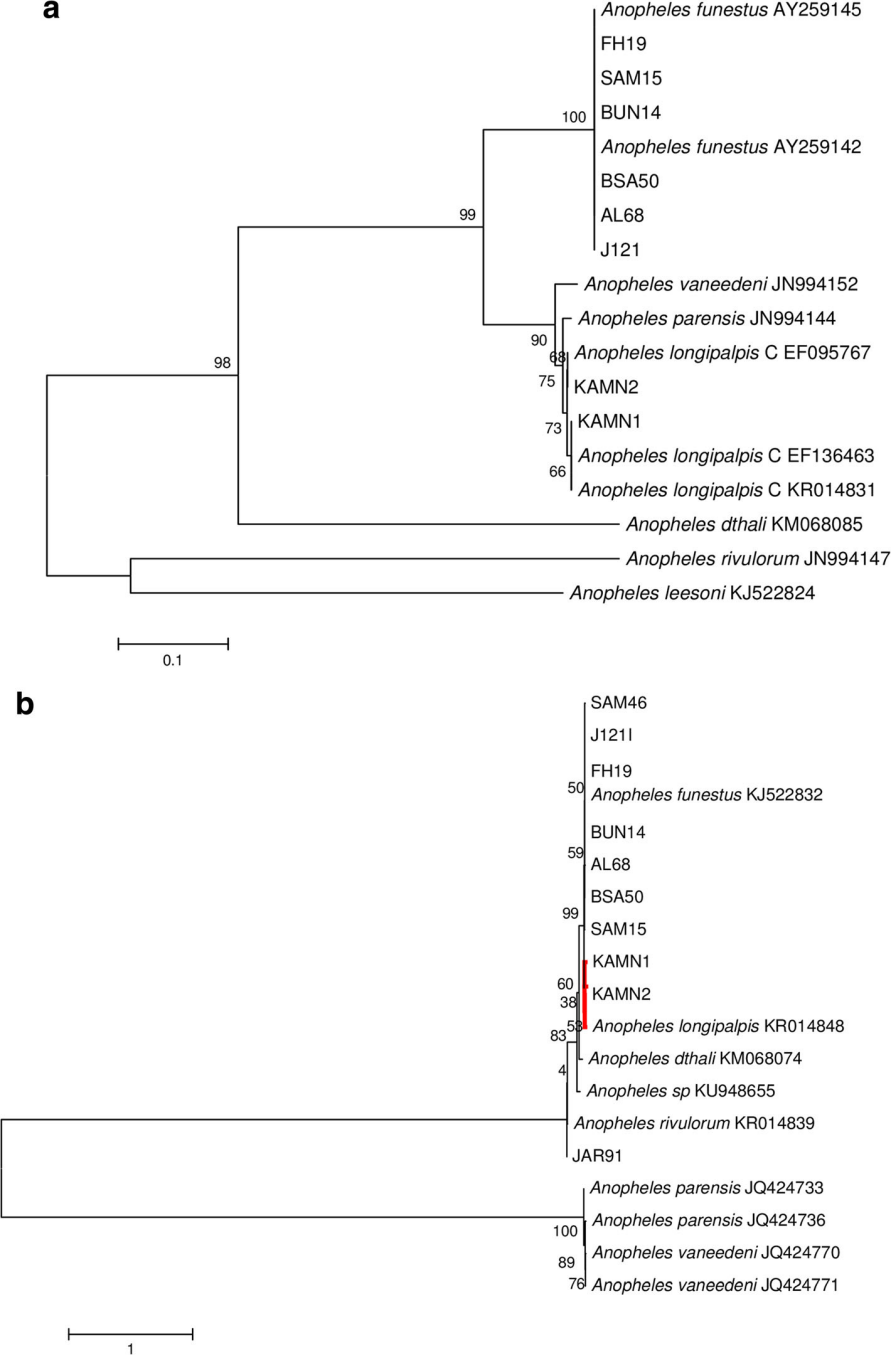
Phylogenetic trees for *Plasmodium* positive mosquito specimens in the *An. funestus* group inferred by applying maximum likelihood analysis in MEGA v.6 based on **a** ITS (267–763 bp), using Kimura-2 model and **b** *cox*1 barcode region (615–658 bp) using GTR+G model. Bootstrap support values from 1000 replications are indicated above nodes. *Anopheles dthali* was included for outgroup purposes. The branch length scale represents substitutions per site. Furthermore, selected sequences of *Plasmodium-*positive specimens were deposited in GenBank [accession numbers: *An. funestus*, MH299885-MH299890 (*cox*1), MH298707-MH298752 (ITS2); *An. rivulorum*, MH547425 (*cox*1); *An. longipalpis*, C MH547426-MH547427 (*cox*1), MH536653-MH536654 (ITS2)]

Among *cox*1 gene (615–658 nt) and/or ITS2 rDNA (267–763 nt) PCR product sequences of specimens found positive for *Plasmodium* infection, 62 shared 100% identity with *An. funestus* [GenBank: KJ522832 (*cox*1); KJ522816 (ITS2)], one shared 100% identity with *An. rivulorum* [GenBank: KR014839 (*cox*1)] and two shared 100% identity with *An. longipalpis* C [GenBank: KR014831, EF095767 (ITS); KR014848 (*cox*1)]. The mosquito specimen identifications are further supported by ML phylogeny (Fig. 3a, b).

Selected sequences of *Plasmodium* isolates detected by the *cox*1 gene (369–477 nt) were deposited in the GenBank database under the accession numbers (MH547428-MH547442 (*P. falciparum*), MH547443 (*P. ovale wallickeri*) and MH547444 (*P. malariae*). Furthermore, selected sequences of *Plasmodium-*positive specimens were deposited in GenBank [accession numbers: *An. funestus* MH299885-MH299890 (*cox*1), MH298707-MH298752 (ITS2); *An. rivulorum* MH547425 (*cox*1), *An. longipalpis* C MH547426-MH547427 (*cox*1), MH536653-MH536654 (ITS2)].

Fifty-nine, two, and one specimens found infected with *P. falciparum*, *P. ovale* and *P. malariae*, respectively, were associated with *An. funestus.* This translated to a *Plasmodium* infection rate (IR) of 9.7% (62/636). One *P. falciprum* infection was associated with *An. rivulorum* (IR = 0.3%, 1/344) and two with *An. longipalpis* C (IR = 28.6%, 2/7). The two *Plasmodium* infected *An. longipalpis* C were collected indoors (2/7) from Kamnarok (Baringo County), while the infective *An. rivulorum* was recorded outdoors (1/253) in Jaribuni from coastal Kenya. *Plasmodium falciparum* infections, especially in *An. funestus*, occurred in diverse sites: *P. ovale* from West Alego and Bunyala, *Plasmodium malariae* from Samia, western Kenya (Fig. 1). Site differences were observed with *Plasmodium* rates ranging from 3.5% in Bunyala to 50% in Lwanya-Bumala; however, these figures need to be interpreted with caution since sampling dates and seasons were variable, hence direct comparisons could not be made. Notably, *Plasmodium* infections were more frequently detected in the malaria endemic western Kenya sites than other sites (Table 1).

**Fig. 4:**
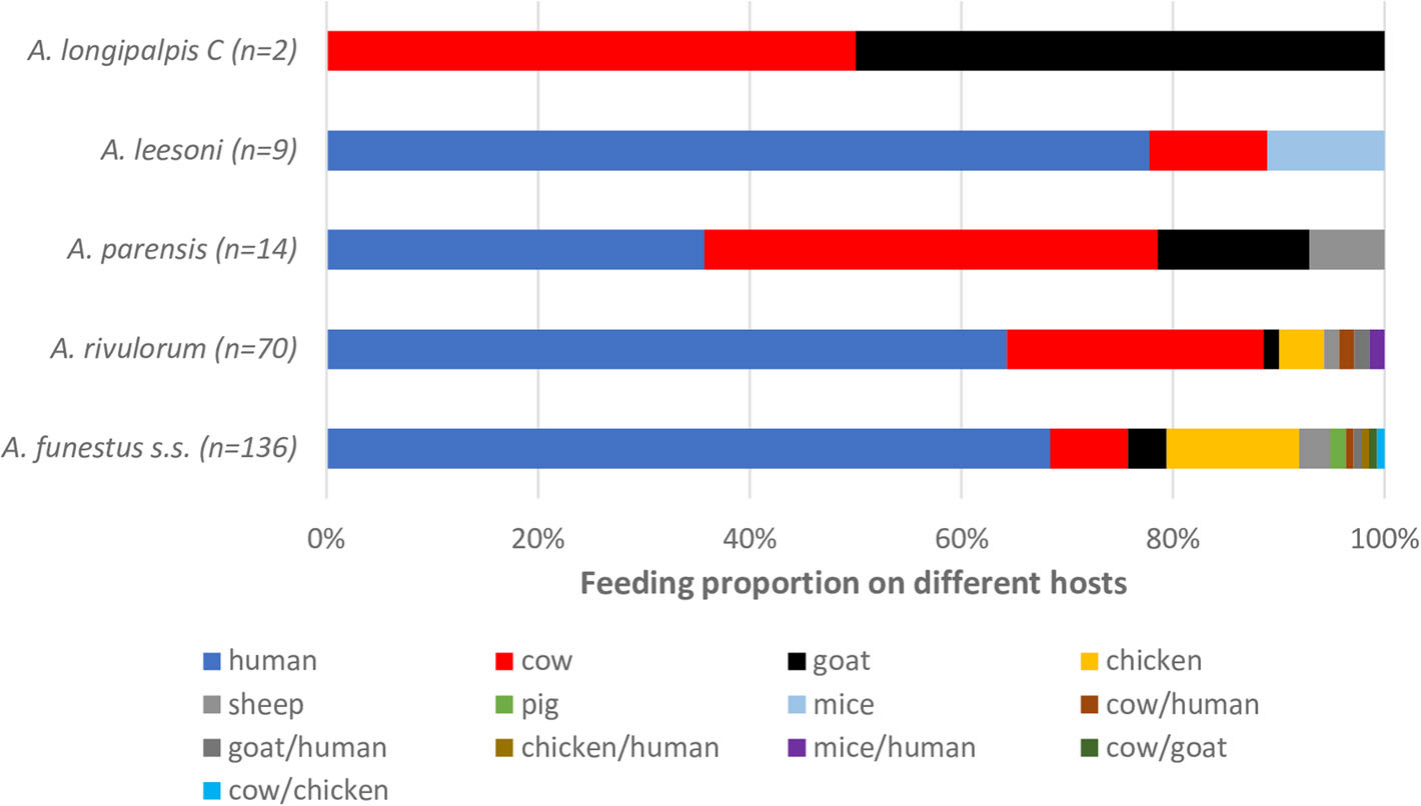
Host blood meal sources for the sibling species in the *Anopheles funestus* group, Kenya. The numbers in parentheses indicates total sample size for each species analyzed

We analyzed 231 engorged specimens for blood-host sources, comprising 48 collected indoors by PSC/aspiration and 183 outdoors using CDC-light trap. Overall, the human blood index for *An. funestus* was 68.4% (93/136) and 64.3% (45/70) for *An. rivulorum*. Human feeds were also encountered for *An. leesoni* and *An. parensis.* The two blood-fed *An. longipalpis* C specimens had each fed on cow and goat. Mixed feeding on humans and other animal hosts and was observed for *An. funestus* and *An. rivulorum*, both of which displayed a feeding tendency on a broad host range (Fig. 4).

## Discussion

Most malaria endemic countries including Kenya have embraced the global plan to eliminate the disease through upscaling of vector control measures, particularly with LLINs [25]. However, these measures have impacted vector behaviors, bionomics and vectorial systems [26, 27]. Our data indicate high *Plasmodium* infection rates in *An. funestus*, affirming its role as the main vector relative to the other sibling species in the group (reviewed in [5]). Furthermore, we provide the first report of *An. longipalpis* C for Kenya. This species, known for its zoophagic but endophilic tendencies has never been associated with *Plasmodium* transmission [28]. However, we found two out of seven specimens *P. falciparum*-positive, indicating that feeding on humans had taken place. Although recorded in low numbers, its potential role as secondary vector in the more arid areas of Kenya should be further investigated.

We uncovered a higher species richness among the *An. funestus* group than previously documented in Kenya. Furthermore, sympatric occurrence of more than three sibling species in a locality as observed in this study, highlights the importance of integrating molecular approaches in surveillance efforts for adequate assessment of the impact of interventions on vector populations [29]. A lack of amplification of some specimens calls for further improvement of existing protocols to adequately discriminate and identify *An. funestus* group members.

We found considerable site differences in *Plasmodium* infection rates in *An. funestus* ranging from 3.5% in Bunyala to 50% in Lwanya-Bumala (Table 1). However, these figures should be interpreted with caution since sampling dates and seasons were variable, hence direct comparisons could not be made. Notably, *Plasmodium* infections were more frequently detected in this species in the malaria endemic western Kenya sites compared to coastal sites. Such site-to-site variation in infection rates is well recognized and may result from differences among populations and their impact on malaria transmission in different ecologies. Differences have been observed at the genetic level between coastal and western populations of *An. funestus* [30] although whether this pattern potentially impacts differentially on transmission has not been assessed. Nonetheless, the high infection rates observed in western Kenya where malaria persists despite indoor vector control [2] warrants further research.

Residual transmission has emerged as a major threat to effective malaria control, as key vectors alter their phenotypes to render current proven vector control interventions such as LLINs less effective. Examples include exhibition of behavioral plasticity in resting and feeding patterns. We found a rather low human blood index (64.8%) for *An. funestus* compared to the near exclusive human feeding habit previously noted for this species [6, 12, 31]. Although variation in this pattern by site or ecology cannot be discounted, the data indicates a certain degree of zoophily and an unusual behavioral divergence in its feeding habits supported by feeding on a broad host-range. Its outdoor behavior might reduce the impact of LLINs on the species and hence contribute to residual transmission. This assertion is supported by its growing prominence in malaria transmission in recent times in Kenya [32] following the dwindling importance of *Anopheles gambiae* (*s.s.*) at large in East Africa [32], which calls for further research into its adaptive biology and role in the disease persistence and even resurgence.

Species records are based on sampling efforts, which in our study was limited in scope (i.e. intensity and duration of trapping in each location) and targeted the rainy seasons only. Seasonal occurrence including species succession has been shown to influence changes in sibling species composition [33]. Studies over longer periods covering seasonal scales need to be encouraged to establish the full profile of sibling species in this group in the context of persistent malaria transmission in Kenya and elsewhere.

Our findings underscore the need for improved surveillance of vector populations employing molecular techniques including sequencing to improve malaria vector control and working towards malaria elimination. This is essential for fine-tuning control strategies in the event of changing local conditions [3] and for discerning cryptic vectors that may contribute to stealth transmission.

## Conclusions

Our findings have revealed a higher species richness among the *An. funestus* group than previously documented in Kenya. Notably is the occurrence of *An. longipalpis* C and for the first time, naturally infected with *Plasmodium falciparum. Anopheles funestus* remains the main vector species in the Funestus group in Kenya which was found to exhibit a rather low human blood index. Our findings underscore the importance of active surveillance through application of molecular approaches to unravel novel parasite-vector associations possibly contributed by cryptic species with important implications for effective malaria control and elimination.

## Additional file

Additional file 1: **Figure S1.** Sequence alignment of 162 nucleotide (nt) *Plasmodium* ncMS sequences corresponding to nucleotide positions 5759–5920 of GenBank accession CP017005. Study sequences are highlighted in bold and GenBank accession numbers and species identifications are indicated for each ncMS gene sequence. In sequence alignment, red = A, green = T, yellow = G, blue = C. grey = nt identity with first sequence. (TIF 503 kb)

## Abbreviations

ASP: Aspiration
CDC: Centers for Disease Control and Prevention
COX1: Cytochrome c oxidase subunit 1
HRM: High resolution melting
LLIN: Long-lasting insecticidal net
PCR: Polymerase chain reaction
PSC: Pyrethrum spray catches
s.l: sensu lato
s.s: sensu stricto
SSA: sub-Saharan Africa
