# Supplementary figures and images for: Insights into malaria transmission among Anopheles funestus mosquitoes, Kenya
Source: Parasit Vectors. 2018 Nov 6;11:577. doi: 10.1186/s13071-018-3171-3 (PMC6219006; doi:10.1186/s13071-018-3171-3)

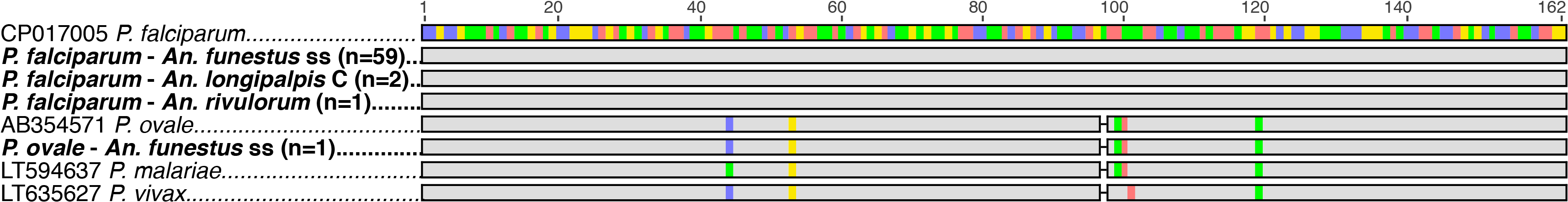

Supplement: Supplementary file 1 — Figure S1. Sequence alignment of 162 nucleotide (nt) Plasmodium ncMS sequences corresponding to nucleotide positions 5759–5920 of GenBank accession CP017005. Study sequences are highlighted in bold and GenBank accession numbers and species identifications are indicated for each ncMS gene sequence. In sequence alignment, red = A, green = T, yellow = G, blue = C. grey = nt identity with first sequence. (TIF 503 kb) [file 13071_2018_3171_MOESM1_ESM.tif]
